# Small Area Forecasting of Opioid-Related Mortality: Bayesian Spatiotemporal Dynamic Modeling Approach

**DOI:** 10.2196/41450

**Published:** 2023-02-10

**Authors:** Cici Bauer, Kehe Zhang, Wenjun Li, Dana Bernson, Olaf Dammann, Marc R LaRochelle, Thomas J Stopka

**Affiliations:** 1 Department of Biostatistics and Data Science School of Public Health The University of Texas Health Science Center at Houston Houston, TX United States; 2 Department of Public Health University of Massachusetts Lowell Lowell, MA United States; 3 Office of Population Health Department of Public Health The Commonwealth of Massachusetts Boston, MA United States; 4 Department of Public Health and Community Medicine Tufts University School of Medicine Boston, MA United States; 5 Department of Gynecology and Obstetrics Hannover Medical School Hannover Germany; 6 Clinical Addiction Research and Education Unit, Section of General Internal Medicine Department of Medicine Boston University School of Medicine Boston, MA United States; 7 Grayken Center for Addiction Boston Medical Center Boston, MA United States; 8 Department of Urban and Environmental Policy and Planning Tufts University Medford, MA United States; 9 Department of Community Health Tufts University Medford, MA United States

**Keywords:** opioid-related mortality, small area estimation, spatiotemporal models, Bayesian, forecasting

## Abstract

**Background:**

Opioid-related overdose mortality has remained at crisis levels across the United States, increasing 5-fold and worsened during the COVID-19 pandemic. The ability to provide forecasts of opioid-related mortality at granular geographical and temporal scales may help guide preemptive public health responses. Current forecasting models focus on prediction on a large geographical scale, such as states or counties, lacking the spatial granularity that local public health officials desire to guide policy decisions and resource allocation.

**Objective:**

The overarching objective of our study was to develop Bayesian spatiotemporal dynamic models to predict opioid-related mortality counts and rates at temporally and geographically granular scales (ie, ZIP Code Tabulation Areas [ZCTAs]) for Massachusetts.

**Methods:**

We obtained decedent data from the Massachusetts Registry of Vital Records and Statistics for 2005 through 2019. We developed Bayesian spatiotemporal dynamic models to predict opioid-related mortality across Massachusetts’ 537 ZCTAs. We evaluated the prediction performance of our models using the one-year ahead approach. We investigated the potential improvement of prediction accuracy by incorporating ZCTA-level demographic and socioeconomic determinants. We identified ZCTAs with the highest predicted opioid-related mortality in terms of rates and counts and stratified them by rural and urban areas.

**Results:**

Bayesian dynamic models with the full spatial and temporal dependency performed best. Inclusion of the ZCTA-level demographic and socioeconomic variables as predictors improved the prediction accuracy, but only in the model that did not account for the neighborhood-level spatial dependency of the ZCTAs. Predictions were better for urban areas than for rural areas, which were more sparsely populated. Using the best performing model and the Massachusetts opioid-related mortality data from 2005 through 2019, our models suggested a stabilizing pattern in opioid-related overdose mortality in 2020 and 2021 if there were no disruptive changes to the trends observed for 2005-2019.

**Conclusions:**

Our Bayesian spatiotemporal models focused on opioid-related overdose mortality data facilitated prediction approaches that can inform preemptive public health decision-making and resource allocation. While sparse data from rural and less populated locales typically pose special challenges in small area predictions, our dynamic Bayesian models, which maximized information borrowing across geographic areas and time points, were used to provide more accurate predictions for small areas. Such approaches can be replicated in other jurisdictions and at varying temporal and geographical levels. We encourage the formation of a modeling consortium for fatal opioid-related overdose predictions, where different modeling techniques could be ensembled to inform public health policy.

## Introduction

Opioid-related overdoses continue to be at crisis levels in communities across the United States, with more than 75,673 fatal overdoses in the 12-month period ending in April 2021 [1-7], worsening during the COVID-19 pandemic [8]. Opioid-related deaths increased more than 5-fold in Massachusetts between 2000 and 2016, with more than 2000 per year from 2016 to 2021 [9,10]. Fatal opioid-related overdose rates above the national level have been ubiquitous across communities in Massachusetts [11]. Despite this crisis, public health responses to the opioid overdose epidemic have been limited by an inability to rapidly identify current fatal overdose patterns, predict future local clusters, and evaluate the effectiveness of interventions.

Identification and prediction of local fatal opioid overdoses require a comprehensive and high-quality surveillance system that provides data sources to capture granular geographic information of the fatal opioid overdose cases across space and time. Ideally, additional information such as individual demographics, past medical history (particularly mental health history), local drug supply, and other risk factors should be included as well to enhance our understanding of the opioid crisis. Many states have established surveillance systems to monitor opioid-related morbidity and mortality to inform planning and evaluate control efforts. Some surveillance systems include unlinked individual data sources for different opioid-related reporting (eg, vital records and prescription drug monitoring programs), and some aim to provide an individually linked database across various data sources [12]. While the latter provides much enhanced data capacity for a wide range of opioid-related research, creating such linked databases takes substantial time and financial resources.

Accurate identification and prediction of fatal opioid-related overdose trends also requires sophisticated spatial and predictive analytical approaches, as noted in a recent review of methodological approaches for the prediction of opioid use–related epidemics in the United States [13]. Most published literature has focused on the identification of community- or neighborhood-level risk factors for opioid-related overdose. For example, Bozorgi et al [14] explored different machine learning and spatial analytical approaches to identify leading contextual risk factors for drug overdose at the block group level in South Carolina. A similar study by Schell et al [15] also focused on identifying new neighborhood-level predictors of opioid-related overdose deaths in Rhode Island at the census block group level, using least absolute shrinkage and selection operator and random forest algorithms. Abell-Hart et al [16] identified counties with a high number of underserved opioid overdose patients in New York state. Basak et al [17] detected spatiotemporal hot spots (ie, counties) at high risk of prescription opioid misuse and overdose using regression models that relied on Medicare claims data in Virginia, North Carolina, and West Virginia.

Statistical models or machine learning techniques proposed specifically for prediction purposes are limited, with most previous studies performed at the US county or state level [18,19], lacking the spatial granularity needed to guide local public health departments for preemptive actions. Bayesian spatiotemporal models have received substantial attention in the past several years in opioid-related research, given their ability to include temporal and spatial correlations and improved precision in small area estimation [20-22]. Sumetsky et al [20] for instance, developed a Bayesian logistic growth model for opioid overdose mortality predictions for 146 counties in North and South Carolina.

In this study, we developed and validated several Bayesian spatiotemporal dynamic predictive models designed for small area forecasting of opioid-related overdose mortality at the ZIP Code Tabulation Area (ZCTA) level. We investigated the benefits of including various area-level demographic and socioeconomic factors in improving the prediction. The predictive performance was assessed using opioid-related overdose mortality data from Massachusetts between 2005 and 2019. We identified the top ZCTAs with the highest predicted fatal opioid overdose rates or counts and by urban and rural areas. Our prediction results can help inform local public health departments’ planning and targeting of resource allocations.

## Methods

### Opioid-Related Mortality Data

Opioid-related mortality data were obtained from the Massachusetts Department of Public Health’s (MDPH’s) Registry of Vital Records and Statistics (RVRS) for 2005 through 2019. International Classification of Disease, Tenth Revision, codes for mortality were used to select from the *underlying cause of death* field by RVRS staff and identify poisonings or overdoses: X40-X44, X60-X64, X85, and Y10-Y14. All multiple *underlying cause of death* fields were then used to define opioid-related death with T40.0, T40.1, T40.2, T40.3, T40.4, and T40.6. We excluded opioid-related deaths for individuals younger than 20 years, since fatal opioid-related death was rare in this age group (n=191). This age grouping also allows for comparison of opioid-related overdose rates with other drug overdose studies [23]. We used the ZCTA of the reported residential address for decedents at the time of the fatal overdose and excluded those either with missing addresses or those with addresses outside of Massachusetts’ boundaries (n=642). The final analytic sample included 16,377 fatal opioid-related overdoses from 2005 to 2019. The flowchart for deriving the analytic samples used for this analysis, summarized by each year, is presented in Figure S1 in Multimedia Appendix 1. We also developed a web-based dashboard to present the map and summaries of these data [24]. Requests for the opioid-related decedent data should be made directly to the MDPH’s RVRS.

### ZCTA Demographic and Socioeconomic Factors

ZCTA-level demographic and socioeconomic variables were obtained from 2005 to 2019 from the American Community Survey 5-year estimates (2005-2009, 2010-2014, and 2015-2019, respectively) [25]. We used the total population counts and characteristics for people aged 20 years or older at the ZCTA level to account for differences in population size. The ZCTA-level demographic variables included race or ethnicity (proportions of White and Hispanic individuals), education (proportion of individuals with a bachelor’s degree), employment (proportion of unemployed individuals), poverty level (proportion of individuals living under the federal poverty level), income (per capita income in US $), living conditions (proportion of renters), transportation (proportion of individuals without vehicles), and English speaking (proportions of individuals with limited English). A detailed summary of these covariates, for all ZCTAs and those stratified by urban or rural areas, is presented in Table S1 in Multimedia Appendix 1. These covariates were considered from a larger set of community-level covariates and were selected on the basis of the literature and our prior research. Following calculation of descriptive statistics and bivariate analyses, we further narrowed the list to a subset of population-level measures at the ZCTA level. The final set of covariates included in this analysis was chosen after fitting a simple Poisson regression model and selecting those with a significant association with opioid-related overdose mortality (see Figure S5 in Multimedia Appendix 1).

### Urban and Rural ZCTAs

The Massachusetts State Office of Rural Health uses a composite scoring system to categorize rurality for each ZCTA. Among the 537 ZCTAs included in this analysis, 390 (73%) were classified as urban areas, and the rest as rural areas. A map of the ZCTAs, color-coded by their urban or rural status, is provided in Figure S2 in Multimedia Appendix 1.

### Statistical Models

Our choice of dynamic spatiotemporal model was motivated by a previous study of drug overdose rates across US counties, which showed that the strongest predictor of overdose rates was overdose rates in nearby counties from the previous year. We considered the following Bayesian dynamic spatiotemporal models [26,27]. *Y_it_* denotes the number of opioid-related fatal overdose counts in ZCTA *i* (*i*=1, …, *I*=537) and year *t* (*t*=2005, …, *t*=2019). We assumed a Poisson distribution with mortality rate *μ_it_*; that is, *Y_it_*|*μ*_it_ ~ *Poisson*(*N_it_μ_it_*), with *N_it_* representing the population size as an offset. The mortality rate *μ_it_* was then decomposed using the following models:

Model 1: log (*μ_it_*) = *α_i_* + *S*(*t*) + *η_t_*, *η_t_* ~ *AR*(1),

Model 2: log (*μ_it_*) = *α_i_* + *S*(*t*) + *ω_it_*, *ω_it_* ~ *GMRF*(τΣ*_AR_*_(1)_ ⊗︀*Σ_I_*), and

Model 3: log (*μ_it_*) = α + *S*(*t*) + *ω_it_*, *ω_it_* ~ *GMRF*(τΣ*_AR_*_(1)_ ⊗︀*Σ_CAR_*)

In all 3 models, *S*(*t*) captured the overall (or marginal) temporal trend and could be modeled via linear or quadratic terms or spline functions. The major differences among the 3 models were how random effects (*η* or *ω*) were modeled in the spatiotemporal interactions. In model 1, *α_i_* was the random effect accounting for the difference of the opioid-related mortality at the ZCTA level, and *η_t_* was a first-order autogressive latent effect *AR*(1) for the temporal trend. Essentially, model 1 regressed the opioid-related mortality rate at time *t*, on the log scale, over the previous time point *t*–1; however, instead of running one model for each ZCTA, the Bayesian framework allowed pooling of the ZCTAs to improve prediction. In models 2 and 3, *α* was the overall intercept, and the ZCTA-level differences of the opioid-related mortality rates were absorbed in the spatiotemporal interaction term *ω_it_*, which now accounted for the spatial dependency between ZCTAs. Models 2 and 3 were inseparable spatiotemporal models, where the interaction term *ω_it_* had a Gaussian Markov Random Field (GMRF) with mean 0 and covariance matrix *τΣ_T_*⊗︀*Σ_S_* [28]. The interaction term was the Kronecker product of the temporal structure *Σ_T_* and the spatial structure *Σ_S_*, and *τ* was the precision parameter (ie, reciprocal of the variance parameter). We again used the *AR*(1) temporal structure in models 2 and 3 but considered different structures for the spatial dependency. In model 2, the spatial structure was an *I*×*I* identity matrix. Although the identity matrix may appear to assume independence of the areas, the hierarchical structure of the model imposed borrowing information from the neighboring areas. In model 3, we assumed a conditional autoregressive model (CAR) [29] as the spatial structure, which assumed that ZCTAs that were geographically adjacent were more similar than those that were far away. We note that other spatial or temporal structures (eg, random walk models) can also be considered in the proposed model framework.

Models 1, 2, and 3 were referred to as the respective base models. We then added ZCTA-level demographic and socioeconomic variables *x*_it_ and the urban or rural indicator variable described above, to each of the base models to assess potential improved prediction performance. All models were fit in a Bayesian framework, with the priors chosen as the noninformative priors commonly used in spatiotemporal models [28]. In our application, it turned out that the interaction term was sufficient to capture the spatial-temporal variation, so we dropped the marginal temporal term. We used the posterior median and 90% credible intervals (CrIs) for inference. The 90% CrI was preferred over the commonly used 95% CrI, as the former was better suited for prediction purposes to avoid overly wide uncertainty. All analyses were performed in RStudio [30] and R package integrated nested Laplace approximation [31].

To assess the predictive performance of the models, we used a one-year ahead approach: assuming we observed data from 2005 to year *t*, we predicted the opioid-related mortality rates and counts for year *t*+1. We started by considering the observed data from 2005 to 2015, and the prediction was carried out for 2016. Then we considered observed data from 2005 to 2016 and predicted counts and rates were calculated for 2017. The one-year ahead prediction procedures were carried out for each year from 2016 to 2019, and the predictions were obtained for each ZCTA, along with the prediction uncertainty (posterior 90% CrI). We used the following metrics to compare the predictive performance across different models: mean absolute error (MAE), root mean square error (RMSE), and the rank difference (RD). MAE was defined as the average absolute difference between the predictive and the observed values; that is, 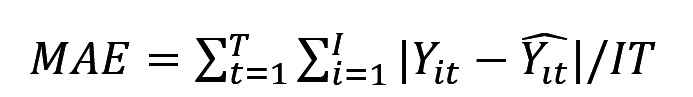, for count or rate *Y_it_*. RMSE was defined as the average squared difference between the predictive and the observed values; that is, 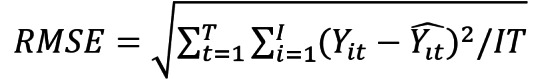. RMSE has the advantage that it was on the same scale as the outcome variable and was thus easy to interpret. For example, if the prediction was for the ZCTA-level mortality count (ie, how many people would die from fatal opioid-related overdose), then an RMSE of 10 would roughly mean that on average the prediction was off by 10 mortality cases. Smaller MAE and RMSE would indicate better performance. The RD was motivated by the correct classification of opioid mortality by group membership, where we divided the ZCTAs into quintiles Q1 to Q5, based on the observed fatal opioid-related overdose rates and counts, and then compared to the quintiles for the predicted rates and counts. The RD was then calculated by the percentage of times when the classification was correct. Higher values of RD indicated better performance.

### Ethical Considerations

This study was reviewed by the Health Sciences institutional review board at Tufts University and was designated as exempt from ethics approval (IRB reference number: 13288).

## Results

The number of fatal opioid-related overdoses among people aged 20 years or older increased from 557 in 2005 to 1912 in 2019, corresponding to an increase in rate from 7.95 in 2005 to 34.4 in 2019 per 100,000 population. Out of a total of 16,377 opioid-related fatalities between 2005 and 2019, overall, 91% occurred in urban ZCTAs. The observed rates at the ZCTA level were highly variable (Figure S3 in Multimedia Appendix 1), ranging from 0 to 1316 per 100,000 population. The extreme values in the observed rates tended to occur in areas with small populations. The high instability of the observed rates, commonly known as the small area estimation problem, poses a special challenge in developing accurate prediction models, which our Bayesian spatiotemporal models helped address.

Results of the predictive performance among the proposed candidate models, using the one-year ahead approach, are presented in Table 1 (for the ZCTA-level fatal opioid-related overdose count) and Table 2 (for the ZCTA-level fatal opioid-related overdose rate). Prediction errors were summarized for all ZCTAs and stratified by rural and urban ZCTAs. Overall, model 3 with the inseparable spatiotemporal interaction term of *AR*(1) and CAR structures performed best for fatal opioid-related overdose predictions, as indicated by smaller MAE and RMSE values. The RMSE showed that the predicted opioid-related death counts, on average, were off only by 2 counts per area. As expected, predictions were better for urban areas than for rural areas, since most of the deaths occurred in urban areas. Addition of demographic and socioeconomic variables generally improved the prediction performance, particularly for model 1; however, the improvement was attenuated in models 2 and 3 where we assumed inseparable spatiotemporal models. The smaller improvement in model 3 was likely because the CAR model already captured the spatial patterns tied to the demographic and socioeconomic factors and hence served as a surrogate of the demographic and socioeconomic variables for predictions. The best performing model using the rank difference showed a less clear pattern, as the values were very similar across the different models. This was particularly true when focusing on the rural ZCTAs, likely due to their small predicted rates or counts and hence very narrow ranges for each quintile.

**Table 1:** Predictive performance assessment of the ZIP Code Tabulation Area (ZCTA) level opioid-related overdose death count, using the root mean and rank difference, and one-year ahead prediction starting with 2016^a^. The smallest root mean square error, mean absolute difference, and highest rank difference for each row are depicted in italics, indicating the best performing models.

Count year		ZCTA type	Model 1		Model 2		Model 3	
			Base Model	Add SDOH^b^	Base Model	Add SDOH	Base Model	Add SDOH
**Root mean square error**								
	2016	All	2.56	2.63	2.98	2.85	*2.35*	2.40
	2017	All	2.06	1.98	2.29	2.20	2.04	*1.94*
	2018	All	2.53	2.47	2.72	2.56	2.47	*2.44*
	2019	All	2.38	2.33	2.57	2.43	2.19	*2.18*
	2016	Urban	2.90	2.98	3.38	3.22	*2.65*	2.70
	2017	Urban	2.34	2.25	2.62	2.50	2.30	*2.20*
	2018	Urban	2.83	*2.75*	3.04	2.84	2.76	2.71
	2019	Urban	2.65	2.59	2.87	2.70	2.46	*2.45*
	2016	Rural	1.26	1.32	1.42	1.43	*1.20*	1.28
	2017	Rural	0.98	*0.97*	1.03	1.03	1.03	0.98
	2018	Rural	1.45	1.45	1.62	1.62	*1.43*	1.48
	2019	Rural	1.42	1.41	1.46	1.47	*1.15*	1.16
**Mean absolute difference**								
	2016	All	0.92	0.98	1.43	1.32	*0.67*	0.74
	2017	All	*0.01*	0.02	0.61	0.50	0.26	0.20
	2018	All	0.55	0.56	0.82	0.73	*0.32*	0.37
	2019	All	0.17	0.19	0.58	0.51	*0.00*	0.06
	2016	Urban	1.15	1.21	1.77	1.60	*0.85*	0.91
	2017	Urban	*0.04*	0.06	0.79	0.63	0.29	0.22
	2018	Urban	0.61	0.60	0.91	0.78	*0.30*	0.35
	2019	Urban	0.13	0.14	0.64	0.52	*0.03*	0.04
	2016	Rural	0.30	0.36	0.52	0.55	*0.22*	0.28
	2017	Rural	0.14	*0.08*	0.13	0.16	0.19	0.13
	2018	Rural	0.40	0.44	0.58	0.60	*0.38*	0.43
	2019	Rural	0.29	0.33	0.43	0.48	*0.06*	0.12
**Rank difference**								
	2016	All	0.50	0.51	0.50	0.49	0.52	*0.53*
	2017	All	0.50	0.50	0.49	0.48	0.51	*0.52*
	2018	All	0.50	0.51	0.50	*0.52*	0.49	*0.52*
	2019	All	0.51	0.51	0.49	0.50	0.50	*0.51*
	2016	Urban	0.54	0.54	0.55	0.54	*0.57*	0.55
	2017	Urban	*0.53*	*0.53*	0.51	0.52	0.52	0.52
	2018	Urban	0.54	0.55	0.55	*0.56*	0.54	0.54
	2019	Urban	0.58	0.60	0.54	0.57	0.61	*0.62*
	2016	Rural	*0.40*	0.39	*0.40*	0.38	0.37	0.35
	2017	Rural	*0.43*	0.42	0.39	0.41	0.41	0.40
	2018	Rural	*0.38*	0.37	0.34	0.36	0.37	0.37
	2019	Rural	0.34	0.37	0.33	0.34	0.37	*0.38*

^a^For example, using data from 2005 to 2015, we predicted the fatal opioid-related overdose count in year 2016 for each of the 537 ZIP Code Tabulation Areas (ZCTAs) in Massachusetts, and compared them to the observed data to assess performance. Similarly, for 2017, we used data from 2006 to 2016, and predicted for 2017. The prediction error was assessed for counts for all ZCTAs and stratified by rural and urban status. Base model refers to Bayesian dynamic spatiotemporal models without any covariates; model results with added covariates of area-level contextual factors are included in the column “Add SDOH.”

^b^SDOH: Social determinants of health.

**Figure 1:**
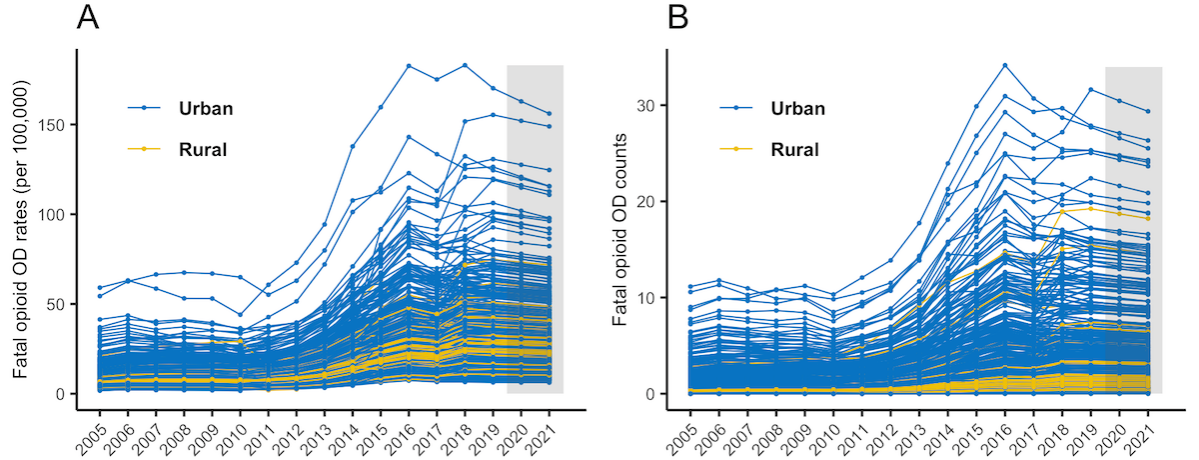
Fitted temporal trends for opioid-related overdose (OD) mortality in Massachusetts between 2005 and 2019, with predictions for 2020 and 2021 (in gray shade). Each line represents a ZIP Code Tabulation Area (ZCTA) color-coded by its urban or rural status. Opioid-related mortality data were obtained from the Massachusetts Registry for Vital Records and Statistics, and predictions were made on the fatal opioid-related overdose rates per 100,000 population (panel A) and count (panel B). These results are from the Bayesian dynamic spatiotemporal Model 3 with ZCTA level demographic and socioeconomic variables.

**Figure 2:**
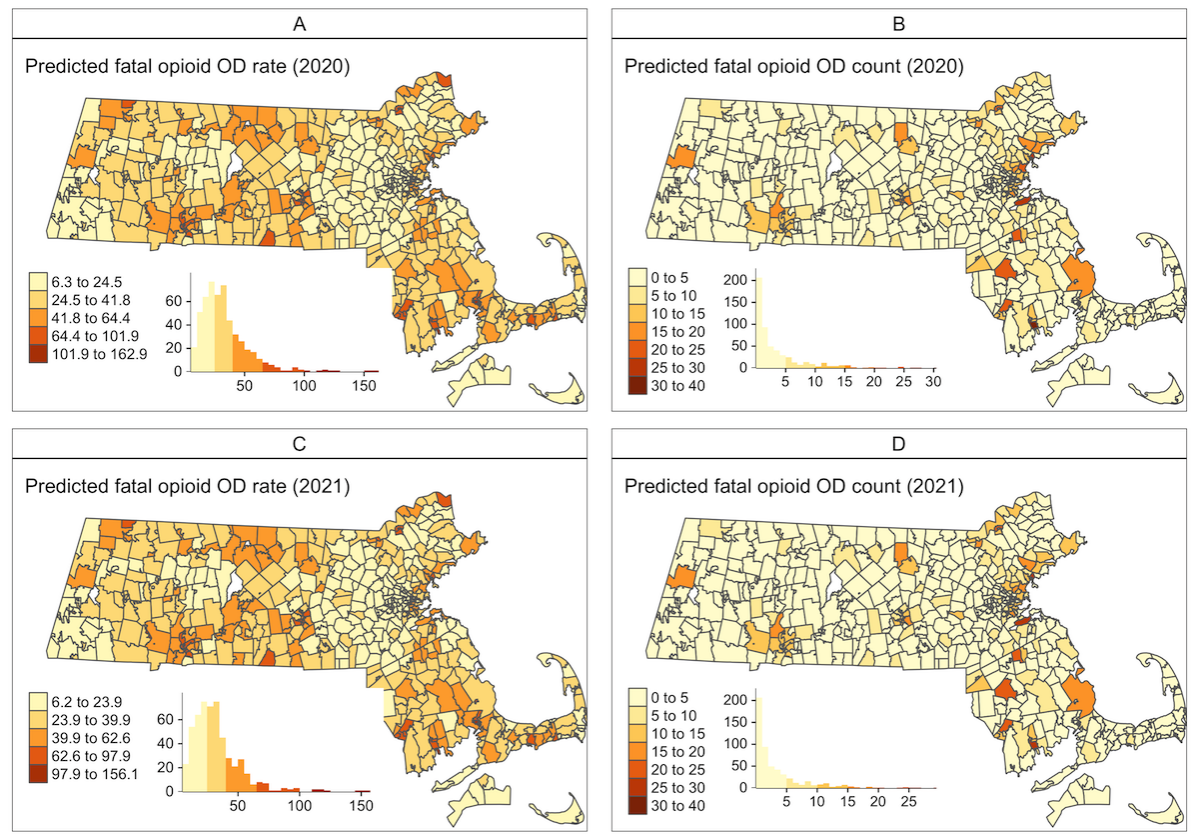
Maps of the predicted ZIP Code Tabulation Area (ZCTA)–level fatal opioid-related overdose (OD) rates (per 100,000 population) and counts for 2020 (A and B) and 2021 (C and D). Data were obtained from Massachusetts Registry for Vital Records and Statistics for 2005 to 2019 and used to predict for 2020 and 2021. These predictions were obtained from the proposed Bayesian dynamic spatiotemporal Model 3 with ZCTA level demographic and socioeconomic variables. In each panel, the embedded histograms present the distribution of the predicted fatal opioid-related overdose rates or counts for that year.

**Figure 3:**
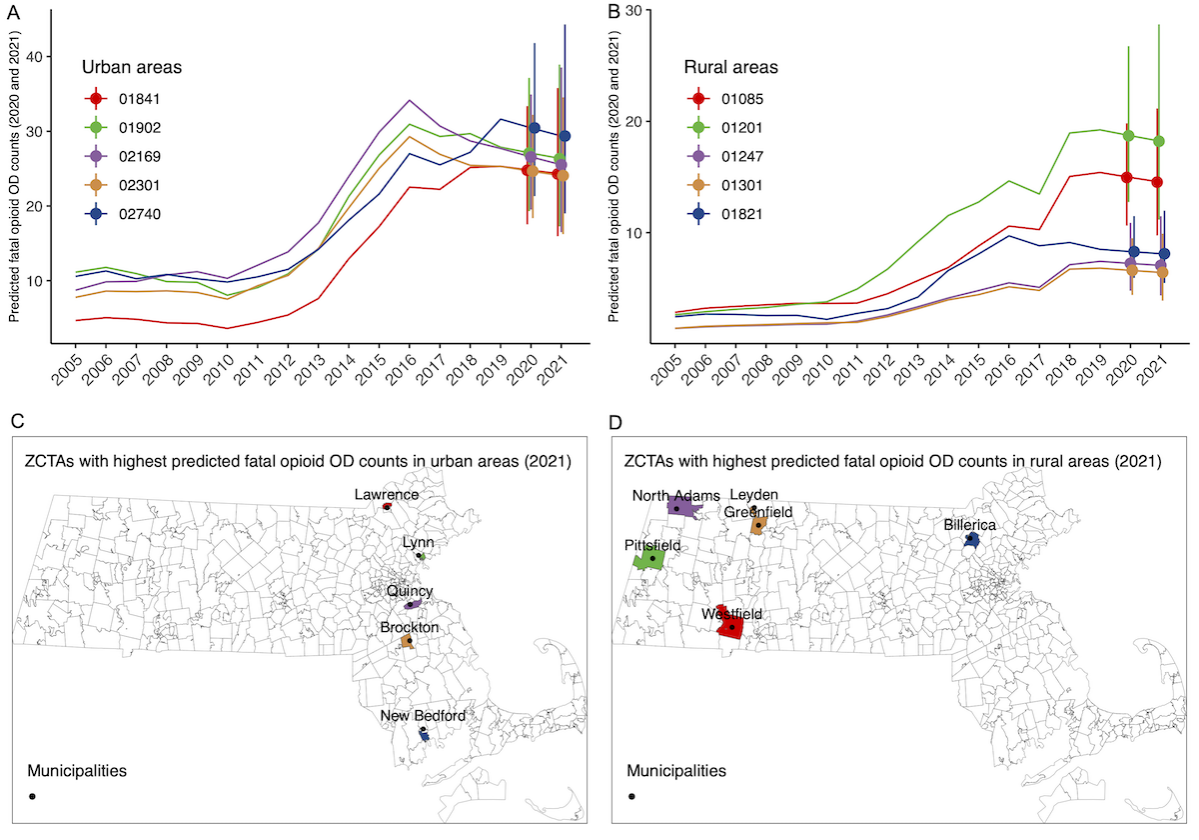
Five selected ZIP Code Tabulation Areas (ZCTAs) with the highest predicted fatal opioid-related overdose (OD) rates or counts for urban (A, C) and rural (B, D) areas. The vertical bars in panels A and B present the 90% posterior credible intervals (CrIs) of the predictions for years 2020 and 2021. Maps (C, D) present the corresponding locations of the identified ZCTAs. Predictions were performed using the proposed Bayesian dynamic spatiotemporal model 3 with ZCTA level demographic and socioeconomic variables and fatal opioid-related overdose data from 2005 to 2019 in Massachusetts.

Since our 1-year prediction assessments suggested that the best performing model was model 3 with the included demographic and socioeconomic variables, we used this model for 2020 and 2021 predictions based on the opioid-related mortality data for 2005-2019. We assumed that ZCTA-level population size and demographic and socioeconomic variables had the same values in 2020 and 2021 as those in 2019. We carried out the prediction for each ZCTA and at the state level. Figure 1 presents the fitted temporal trends of opioid-related mortality in Massachusetts between 2005 and 2019, with predictions carried out for 2020 and 2021 (the gray shaded area), both as the rate per 100,000 population (panel A) and total counts (panel B). Each line represents an individual ZCTA, color-coded by urban or rural status. Figure 2 presents the maps of the predicted opioid-related mortality rates and counts for 2020 and 2021. At the ZCTA-level, the predicted opioid-related mortality rate ranged from 6.11 to 162 per 100,000 population for 2020, and 6.05 to 158 per 100,000 population in 2021. Note that because of the “smoothing” effect in Bayesian models, the estimated or predicted rate could be very close to 0 but would never be exactly 0. Therefore, for ZCTAs that may have zero observed deaths, the predicted rate would not be zero but would be skewed toward the average rate. In addition, any ZCTAs that may have low observed rates but were “surrounded” by ZCTAs with high rates would have higher predicted rates, reflecting how the spatial models borrow information from neighboring ZCTAs. Because of the large variation in population size by ZCTA, the spatial patterns seen in the maps in Figure 2 for predicted rate and counts are quite different, where the latter are generally centered around highly populated areas. The predicted count ranges from close to 0 (after rounding up to integers) to 30 (90% CrI 21-41) for 2020, and 29 (90% CrI 19-44) for 2021. We also identified the top 5 ZCTAs with the highest predicted counts, by urban or rural classifications, and presented the prediction results in Figure 3. The urban ZCTAs were located within cities with high fatal opioid-related overdose risks: Lawrence, Lynn, Quincy, Brockton, and New Bedford (Figure 3C). The rural ZCTAs were located in municipalities that were known to have high risks for fatal opioid-related overdoses, including Pittsfield, North Adams, Greenfield, Westfield, and Billerica (Figure 3D).

At the state level, the predicted opioid-related mortality rate for the population aged 20 years and older was 35.79 per 100,000 population (90% CrI 29.4-43.4) for 2020, and 35.81 (90% CrI 26.9-46.0) for 2021. For urban areas, the predicted rate was 36.0 (90% CrI 29.2-43.7) in 2020, and 35.5 (90% CrI 26.8-46.1) in 2021. For rural areas, the predicted rate was 33.9 (90% CrI 26.6-43.3) in 2020, and 34.6 (90% CrI 25.2-53.4) in 2021. The CrIs for the prediction were wider in 2021 than in 2020 and in rural areas than in urban areas. This was expected as the further ahead we attempted to predict outcomes, the less certainty we would have. Our prediction suggested that the opioid-related overdose death counts for 2020 would be 1887 (90% CrI 1549-2285) for the whole state, with 1682 (90% CrI 1364-2041) in urban ZCTAs, and 203 (90% CrI 160-260) in rural ZCTAs, and those for 2021 would be 1888 (90% CrI 1419-2426) for the whole state with 1656 (90% CrI 1253-2149) in urban ZCTAs, and 208 (90% CrI 151-320) in rural ZCTAs.

**Table 2:** Predictive performance assessment of the ZIP Code Tabulation Area (ZCTA) level opioid-related overdose death rate, using the root mean and rank difference, and one-year ahead prediction starting with 2016^a^. The smallest root mean square error, mean absolute difference, and highest rank difference for each row are depicted in italics, indicating the best performing models.

Rate year			ZCTA type		Model 1					Model 2					Model 3		
					Base Model		Add SDOH^b^		Base Model			Add SDOH		Base Model			Add SDOH
**Root mean square error**																	
	2016	All		41.18		40.27		42.84			41.94		40.28			*38.95*	
	2017	All		29.21		30.18		29.01			30.24		*28.63*			29.82	
	2018	All		40.54		*40.22*		42.63			41.83		40.47			40.65	
	2019	All		74.80		75.64		75.75			76.03		*73.26*			75.39	
	2016	Urban		44.53		43.39		46.52			45.45		43.57			*41.65*	
	2017	Urban		30.31		31.76		30.75			32.30		*29.63*			31.13	
	2018	Urban		38.30		*37.67*		40.82			39.71		38.17			38.24	
	2019	Urban		83.48		84.42		84.48			84.74		*81.59*			84.13	
	2016	Rural		30.50		30.45		30.98			30.74		*29.79*			30.62	
	2017	Rural		26.08		25.53		*23.76*			23.91		25.80			26.02	
	2018	Rural		*46.00*		46.33		47.10			47.03		46.04			46.47	
	2019	Rural		44.05		44.49		44.90			45.31		*43.99*			44.38	
**Mean absolute difference**																	
	2016	All		7.29		7.27		13.73			12.44		*4.91*			4.95	
	2017	All		3.70		4.01		4.31			*2.81*		5.87			5.83	
	2018	All		5.89		5.36		10.59			9.26		4.10			*4.05*	
	2019	All		4.47		4.01		10.07			8.83		1.60			*1.57*	
	2016	Urban		10.02		9.41		16.37			14.41		7.25			*7.01*	
	2017	Urban		*2.11*		3.09		5.69			3.56		5.09			5.24	
	2018	Urban		5.72		4.61		10.05			8.15		3.01			*2.83*	
	2019	Urban		4.60		3.64		10.02			8.24		2.07			*1.91*	
	2016	Rural		*0.00*		1.57		6.68			7.20		1.31			0.55	
	2017	Rural		7.94		6.44		*0.64*			0.84		7.93			7.40	
	2018	Rural		*6.34*		7.39		12.04			12.23		6.98			7.29	
	2019	Rural		4.13		5.02		10.22			10.40		*0.35*			0.68	
**Rank difference**																	
	2016	All		0.30		*0.34*		0.31			0.33		0.31			0.32	
	2017	All		0.32		0.36		0.32			*0.37*		0.34			0.35	
	2018	All		0.29		0.31		0.31			0.30		0.31			*0.34*	
	2019	All		0.33		0.34		0.34			*0.35*		0.34			0.32	
	2016	Urban		0.32		*0.38*		0.31			0.37		0.33			*0.38*	
	2017	Urban		0.34		*0.38*		0.35			0.35		0.37			*0.38*	
	2018	Urban		0.32		0.34		0.30			0.33		0.33			0.34	
	2019	Urban		0.36		0.36		0.36			0.37		*0.38*			0.36	
	2016	Rural		0.28		0.24		*0.30*			0.24		0.29			0.26	
	2017	Rural		0.19		0.21		*0.30*			0.27		0.22			0.23	
	2018	Rural		0.21		*0.26*		0.24			0.24		*0.26*			*0.26*	
	2019	Rural		*0.28*		0.25		0.25			0.26		0.27			*0.28*	

^a^For example, using data from 2005 to 2015, we predicted the fatal opioid-related overdose rate in 2016 for each of the 537 ZIP Code Tabulation Areas (ZCTAs) in Massachusetts, and compared the predicted rates to the observed rates to assess prediction performance. Similarly, for 2017, we used data from 2006 to 2016 and predicted for 2017. The prediction error was assessed for rates for all ZCTAs and stratified by rural and urban status. Base model refers to Bayesian dynamic spatiotemporal models without any covariates; model results with added covariates of area-level contextual factors are included in the column labeled “Add SDOH.”

^b^SDOH: Social determinants of health.

## Discussion

In this analysis, we developed and compared several Bayesian spatiotemporal dynamic models for predicting small area opioid-related mortality. The prediction performance was evaluated with data from the MDPH RVRS for 2005 through 2019 using the one-year ahead approach, with and without ZCTA-level demographic and socioeconomic variables. Using data from 2005 through 2019 and the best performing model, we also predicted the fatal opioid-related death rates and counts for 2020 and 2021, respectively, along with uncertainty assessments. We also identified the ZCTAs—by urban and rural status—that had the highest predicted opioid-related mortality, both by rates and by counts.

Our prediction showed that, if there was no interruptive change to the trends observed for 2005-2019, we would observe a stabling trend of the fatal opioid-related overdose in Massachusetts for 2020 and 2021. The stabling trend would be applicable to the entire state (ie, the state-level total fatal opioid-related overdose) as well as for most ZCTAs in Massachusetts. Our prediction also identified the ZCTAs deviating from this stabilizing trend, with continually increasing rates above the state average. Identifying ZCTAs with the predicted high risks allows for the possibility of preemptive and geo-targeted public health interventions. The prediction models presented here allowed for a more granular depiction of existing and expected trends in opioid-related overdose deaths over spatially granular units, which differed from other existing models developed for predication at larger geographical scales (eg, state or county). Such predictions are instrumental for state and local public health departments’ planning to identify and address potential service gaps for deploying harm reduction and treatment interventions. Local data are largely limited both in type and quantity; drug seizure data, for example, are often not available at more granular levels than the state, limiting input information in predictive modeling. However, up-to-date data at the local scale are instrumental to developing the prediction models and engaging communities in designing and implementing data-driven responses to reduce opioid-related harms [32].

At the time of this analysis, we only obtained the fatal opioid-related overdose data for Massachusetts from 2005 to 2019 and partial data in 2020 (incomplete data with only the first 6 months). We designed our analysis and prediction evaluation on the basis of the data available to us, but we lacked the official statistics for 2020 and 2021 to assess the prediction accuracy for those years. The latest Massachusetts opioid-related overdose data brief released in December 2022 [33] reported the confirmed opioid-related overdose deaths among Massachusetts residents for 2020 and 2021. Although the data brief reported the total number and rate including all ages, which was different from the adult population we focused on, it was clear that we underpredicted the total number of fatal opioid-related overdose in Massachusetts for 2020 and 2021. The data brief also noted a 9% increase in the opioid-related overdose death rate in Massachusetts for 2021 over 2020. However, since the brief only reported the total opioid overdose death counts at the state level and not by ZCTAs, we were not able to assess the underprediction at a more granular spatial level. The sharp increase in the fatal opioid-related overdose was likely due to the substantive impact of the COVID-19 pandemic [8,34], which disrupted any established trends prior to 2020 that informed our prediction modeling. The time lag in the drug overdose database had been identified as a main barrier to developing fatal overdose predictive models, as reported by Borquez and Martin [35]. Our future work, in collaboration with the MDPH, plans to use the statewide Public Health Data warehouse [36] to obtain timelier and rich data sources with local information in order to improve predictive performance in small areas.

It is important to consider the limitations inherent in the highlighted analyses, and recommendations that should be considered when developing future opioid-related overdose prediction models. First, the spatial units of ZCTAs used in our study may not be the ideal spatial unit for prediction, as the population size within ZCTAs vary substantially, compared to other spatial units of analysis (eg, census tracts) where, by design, the population sizes are much more homogenous. In addition, ZCTAs may not be sufficiently granular, from a spatial perspective, to identify local hot spots. However, they are extensively used in spatial analysis as they are most readily available in many aggregated data sources (eg, surveillance or insurance claims databases). ZCTAs provide useful geospatial information for analyses while often also satisfying data privacy concerns. Second, a better understanding of the contributing factors to local opioid overdose trends and patterns, with reliable measures representing such factors, would clearly improve prediction power and accuracy. For example, toxicology data would have helped us to include the appearance of fentanyl in the local drug supply, to aid the prediction in the shifting “waves” in opioid-related fatal overdose [37]. Third, we used the ZCTA of the decedents’ residences, rather than the injury addresses or the locations where the fatal opioid overdoses were recorded. Injury data generally have a high level of missingness (~50% in MA), and the recorded death location, if not at the decedent’s place of residence, is often recorded at a hospital, even though the injury (ie, overdose event) typically occurred elsewhere. Finally, and perhaps most challenging, is to incorporate the impact of emerging phenomena such as the COVID-19 pandemic into predictions of opioid-related overdose trends, which was not possible for our analyses given the data availability at the time of analyses. This task requires the real-time data inputs and requires a joint effort among researchers and practitioners from multiple agencies, institutions, and sectors. We have seen a lot of progress made on this front during the COVID-19 pandemic, and we hope to see more progress in the drug overdose research in the future.

Despite the abovementioned data source and methodological limitations, our models showed promise in providing reasonable 1-year forecasts of opioid-related mortality in MA with geographic granularity using existing data, as the short-term point estimates for the number of overdoses tended to be close to the true value. Our Bayesian spatiotemporal models further demonstrated the advantages of incorporating inseparable spatiotemporal dependencies over the simpler regression models without such dependence. Since the assumed spatial dependency structure captured the spatial patterns tied to many demographic and socioeconomic factors, such models do not rely on the knowledge of the future measures of these factors in predicting opioid-related mortality. Prediction is a challenging problem in general, and though many models have been developed in various contexts, it is almost impossible to find one single model or approach that universally performs best [38]. Although our analysis could not investigate all possible predictive models for fatal opioid-related overdoses, we provided a novel approach to forecasting overdose events for small geographic areas. Compared to other predictive approaches, Bayesian models provide a natural framework where the prediction can be conveniently included in the model fitting process, by treating predictions as missing values. Our findings demonstrated the utility of sophisticated Bayesian spatiotemporal dynamic models in supporting state and local opioid surveillance and the ability to provide prediction at a granular geographic level, offering a unique opportunity for preemptive public health and policy interventions, replacing reactionary public health responses. Echoing Borquez and Martin [35], we encourage the field to consider a modeling consortium for opioid-related prediction models, where different modeling techniques could be ensembled.

## Appendix

Multimedia Appendix 1 Trends of opioid-related deaths in Massachusetts.

## Abbreviations

CAR: conditional autoregressive model
CrI: credible interval
MAE: mean absolute error
MDPH: Massachusetts Department of Public Health
RD: rank difference
RMSE: root mean square error
RVRS: Registry of Vital Records and Statistics
ZCTA: ZIP Code Tabulation Area
