# Supplementary material for: Small Area Forecasting of Opioid-Related Mortality: Bayesian Spatiotemporal Dynamic Modeling Approach
Source: JMIR Public Health Surveill. 2023 Feb 10;9:e41450. doi: 10.2196/41450 (PMC9960038; doi:10.2196/41450)
Supplement: Multimedia Appendix 1 [file publichealth_v9i1e41450_app1.pdf]

Small Area Forecasting of Opioid-related Mortality: A Bayesian Spatiotemporal Dynamic Modeling Approach

Supplementary Materials

Table S1. ZCTA-level summaries of demographic and socioeconomic determinants, overall and stratified by urban/rural status.

Figure S1. Data processing flowchart.

Figure S2. Map of 537 ZCTAs in Massachusetts, color-coded by urban/rural classification.

Figure S3. Observed ZCTA-level opioid overdose fatality rate per 100,000 population from 2005 to 2019.

Figure S4. Maps of the ZCTA-level demographic and socioeconomic variables used in the predictive models.

Figure S5. Demographic and socioeconomic determinants associated with opioid overdose mortality in Massachusetts ZIP Code Tabulation Areas (ZCTAs), 2005-2019 (n=537).

Figures S6-S11. Maps presenting the estimated random effects from the Bayesian spatiotemporal dynamics models used in this analysis. We present the estimated spatiotemporal random effects from the following 6 models: Model 1 "Base Model", Model 1 "Add SDoH"; Model 2 "Base Model", Model 2 "Add SDoH"; and Model 3 "Base Model", Model 3 "Add SDoH".

Figure S12. Estimated temporal random effects in selected areas from the Bayesian spatiotemporal dynamics models used in this analysis.

Table S2. Summary of hyperparameters from fitting the proposed Bayesian dynamic spatiotemporal models.

Table S1. ZCTA-level summaries of demographic and socioeconomic determinants, overall and stratified by urban/rural status. These data were obtained from 5-year American Community Survey (ACS) estimates, by the corresponding timeframes.

	2005-2009			2010-2014			2015-2019		
**Variable**	**All**	**Urban**	**Rural**	**All**	**Urban**	**Rural**	**All**	**Urban**	**Rural**
**Population**									
Mean (CV%)	12200 (98.8%)	14900 (84.7%)	5140 (126.5%)	12500 (99.7%)	15300 (85.3%)	5170 (127.4%)	12800 (100.1%)	15700 (85.7%)	5250 (126.4%)
Median [Q1, Q3]	8460 [2720, 17700]	12700 [3950, 22200]	3410 [1430, 6340]	8570 [2580, 18100]	13000 [4160, 23300]	3410 [1400, 6510]	8690 [2620, 18500]	13400 [4190, 23400]	3580 [1330, 6630]
**White, %**									
Mean (CV%)	84.8 (20.9%)	81.4 (23.9%)	93.7 (5.3%)	83.2 (22.3%)	79.4 (25.5%)	93.2 (6.1%)	81.1 (23.2%)	77.1 (26.5%)	91.8 (6.1%)
Median [Q1, Q3]	91.5 [81.8, 95.5]	88.8 [75.9, 94.0]	94.6 [91.6, 97.0]	90.7 [78.6, 95.2]	85.8 [73.0, 93.4]	95.0 [91.6, 96.6]	87.7 [75.2, 93.4]	83.6 [69.1, 91.7]	92.8 [88.5, 95.9]
**Hispanic, %**									
Mean (CV%)	5.83 (178.1%)	7.17 (164.8%)	2.24 (101.5%)	6.58 (169.8%)	8.08 (156.7%)	2.59 (112.0%)	7.54 (154.6%)	9.22 (143.0%)	3.06 (88.6%)
Median [Q1, Q3]	2.50 [1.10, 5.80]	3.00 [1.40, 7.15]	1.60 [0.70, 3.10]	2.90 [1.30, 6.40]	3.50 [1.80, 8.50]	1.80 [0.85, 3.10]	3.90 [2.00, 7.60]	4.70 [2.40, 9.63]	2.40 [1.23, 4.08]
**Unemployed, %**									
Mean (CV%)	4.91 (54.3%)	5.03 (57.3%)	4.59 (42.5%)	4.72 (54.6%)	4.91 (55.5%)	4.19 (49.0%)	2.91 (59.5%)	3.01 (60.9%)	2.63 (52.5%)
Median [Q1, Q3]	4.70 [3.58, 6.00]	4.70 [3.60, 6.20]	4.60 [3.20, 5.80]	4.40 [3.35, 5.75]	4.50 [3.50, 6.00]	4.30 [2.85, 5.40]	2.70 [1.93, 3.58]	2.70 [2.00, 3.70]	2.45 [1.80, 3.20]
**Bachelor, %**									
Mean (CV%)	40.3 (48.2%)	41.3 (50.5%)	37.7 (38.8%)	41.8 (47.3%)	42.5 (49.6%)	40.0 (39.3%)	44.6 (44.2%)	45.9 (46.2%)	41.2 (35.4%)
Median	36.9	37.7	34.5	38.6	39.5	37.5	42.1	43.6	39.0

	2005-2009			2010-2014			2015-2019		
**Variable**	**All**	**Urban**	**Rural**	**All**	**Urban**	**Rural**	**All**	**Urban**	**Rural**
[Q1, Q3]	[25.9, 52.1]	[25.0, 55.1]	[26.8, 46.4]	[27.2, 53.6]	[26.0, 57.1]	[29.9, 48.3]	[29.8, 57.5]	[28.9, 61.6]	[31.7, 49.8]
Missing	0 (0%)	0 (0%)	0 (0%)	0 (0%)	0 (0%)	0 (0%)	2 (0.4%)	2 (0.5%)	0 (0%)
**Poverty, %**									
Mean (CV%)	6.11 (118.5%)	6.67 (119.4%)	4.65 (98.4%)	6.60 (112.4%)	7.33 (114.2%)	4.69 (70.2%)	5.92 (137.6%)	6.76 (136.1%)	3.72 (93.3%)
Median [Q1, Q3]	3.70 [1.80, 8.00]	3.80 [1.88, 8.43]	3.50 [1.80, 7.00]	4.20 [2.40, 8.10]	4.30 [2.30, 9.10]	4.10 [2.50, 6.50]	3.70 [1.90, 6.80]	3.95 [2.00, 8.13]	3.20 [1.50, 5.10]
Missing	7 (1.3%)	7 (1.8%)	0 (0%)	9 (1.7%)	7 (1.8%)	2 (1.4%)	9 (1.7%)	8 (2.1%)	1 (0.7%)
**Limited English, %**									
Mean (CV%)	5.38 (141.2%)	6.79 (124.3%)	1.64 (101.5%)	5.23 (141.7%)	6.65 (123.4%)	1.50 (118.7%)	5.38 (138.7%)	6.74 (122.7%)	1.77 (115.6%)
Median [Q1, Q3]	2.50 [1.00, 6.63]	3.60 [1.70, 9.15]	1.20 [0.70, 2.20]	2.30 [0.90, 6.65]	3.80 [1.40, 8.53]	0.90 [0.40, 1.90]	2.75 [0.93, 6.78]	3.80 [1.58, 8.50]	1.05 [0.33, 2.30]
**No vehicle, %**									
Mean (CV%)	8.70 (133.2%)	10.40 (125.0%)	4.22 (93.8%)	8.72 (131.2%)	10.40 (122.5%)	4.40 (114.4%)	8.99 (140.4%)	10.90 (130.3%)	3.93 (85.9%)
Median [Q1, Q3]	4.80 [2.30, 9.43]	5.50 [2.80, 12.20]	3.10 [1.30, 5.60]	4.70 [2.50, 9.40]	5.70 [3.10, 12.30]	3.20 [1.70, 5.65]	4.80 [2.40, 9.40]	5.60 [3.10, 12.50]	3.25 [1.53, 5.60]
Missing	4 (0.8%)	4 (1.0%)	0 (0%)	6 (1.1%)	6 (1.5%)	0 (0%)	4 (0.7%)	4 (1.0%)	0 (0%)

Abbreviations: CV, coefficient of variation; Q1: first quantile; Q3: third quantile.

Figure S1: Data processing flowchart.

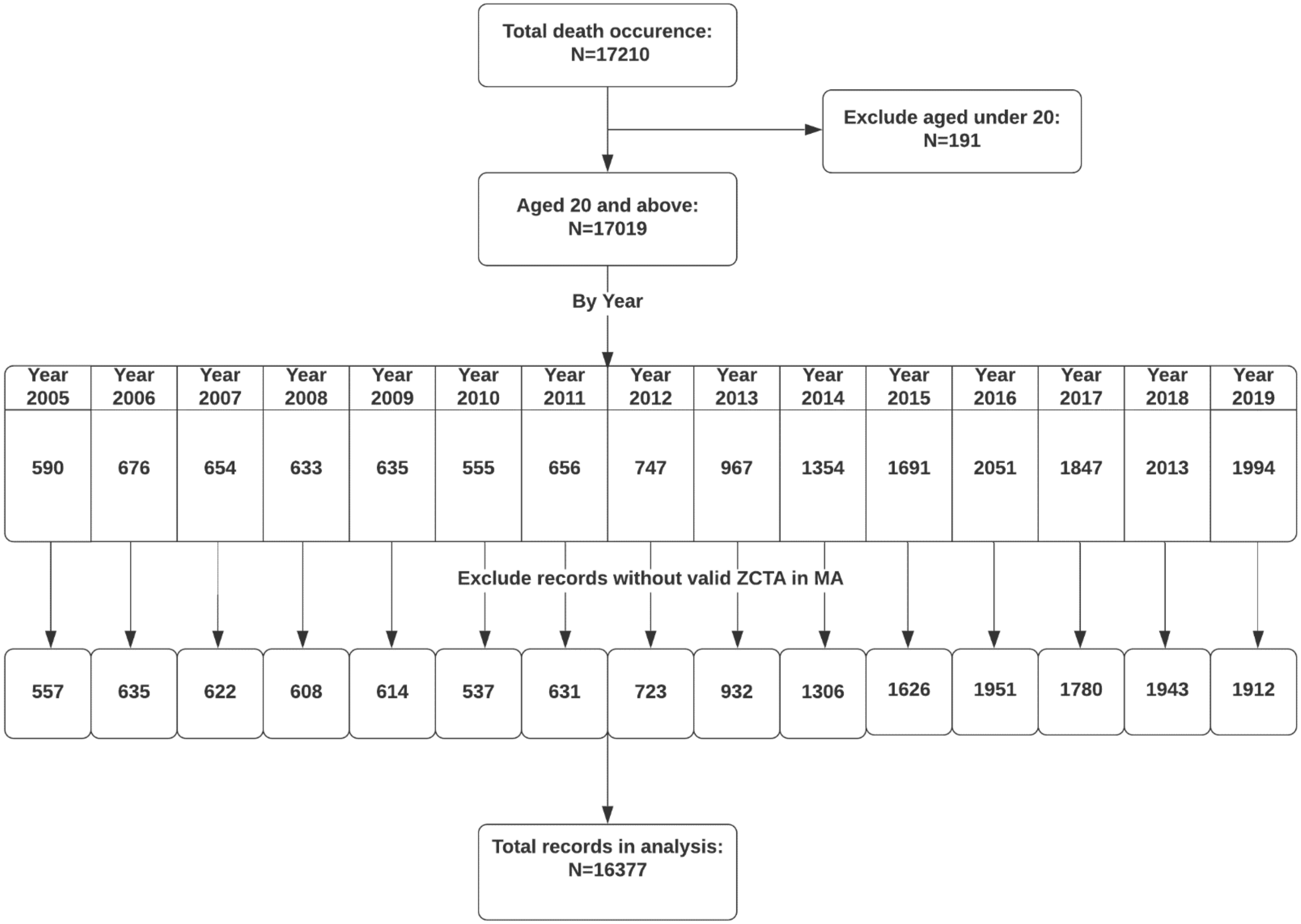

Figure S2: Map of 537 ZCTAs in Massachusetts, color-coded by urban/rural classification. Of these, 390 (73%) are classified as urban areas and 140 (27%) as rural areas in 2019.

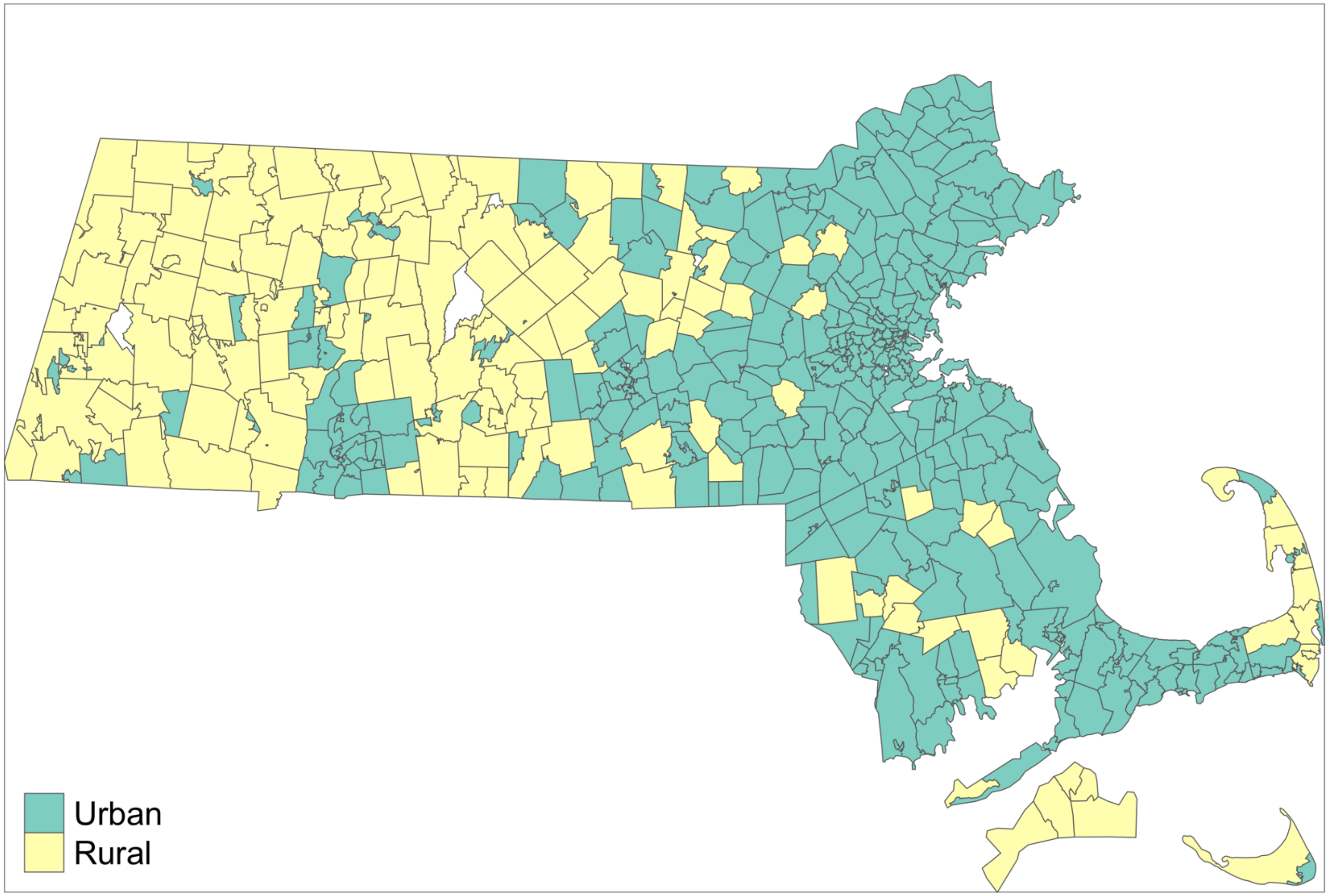

Figure S3: Observed ZCTA-level opioid overdose fatality rate per 100,000 population from 2005 to 2019. Observed rates from ZCTAs with small population sizes had high variation over time, as seen by some extremely large values.

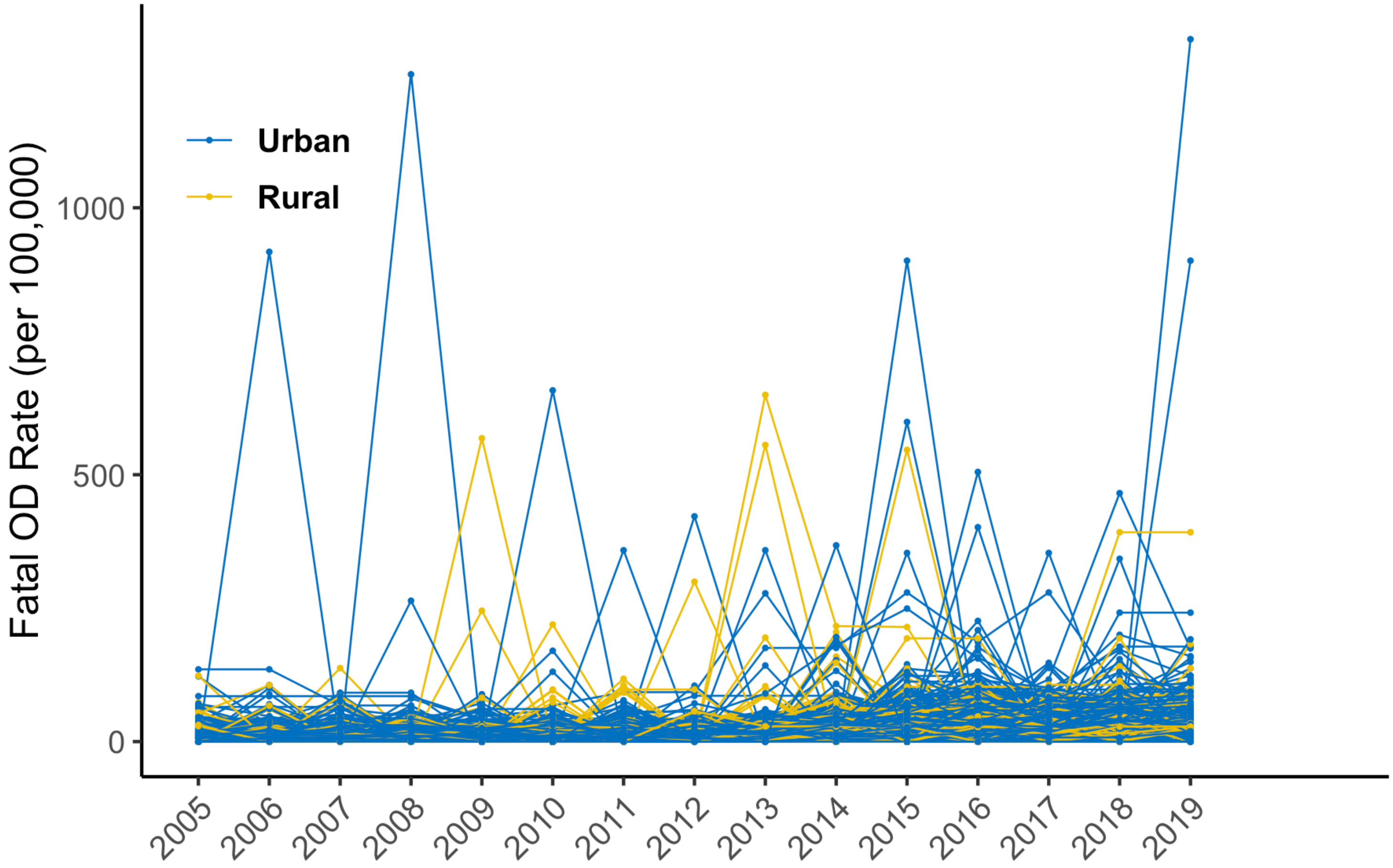

Figure S4: Maps of the ZCTA-level demographic and socioeconomic variables used in the predictive models. Data for these variables are extracted from American Community Survey 5-year estimates for 2015-2019.

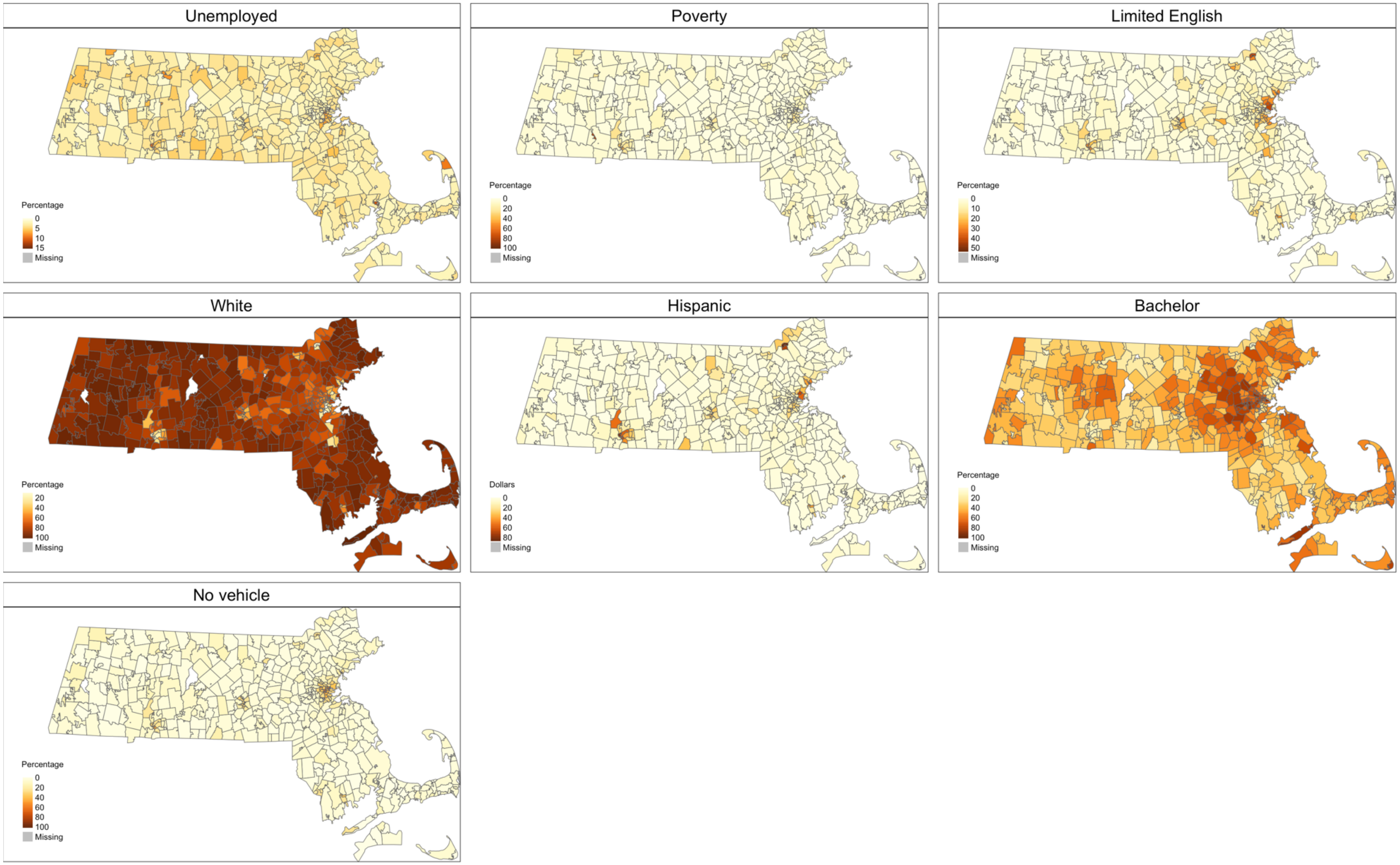

Figure S5. Demographic and socioeconomic determinants associated with opioid overdose mortality in Massachusetts ZIP Code Tabulation Areas (ZCTAs), 2005-2019 (n=537). Results are from fitting a simple Bayesian Poisson model, and presented as the relative risks (RR) and posterior 95% credible intervals (95% CrI).

Opioid Overdose Mortality

ZCTA Level Variable	RR (95% CI)
Urban vs. Rural	1.286 (1.216, 1.361)
Bachelor degree (%)	0.554 (0.540, 0.568)
Unemployment (%)	0.576 (0.562, 0.591)
Living in poverty (%)	1.155 (1.119, 1.191)
White (%)	0.888 (0.866, 0.911)
Hispanic (%)	0.894 (0.876, 0.912)
Limited English (%)	1.014 (0.991, 1.038)
No vehicle (%)	1.114 (1.083, 1.145)

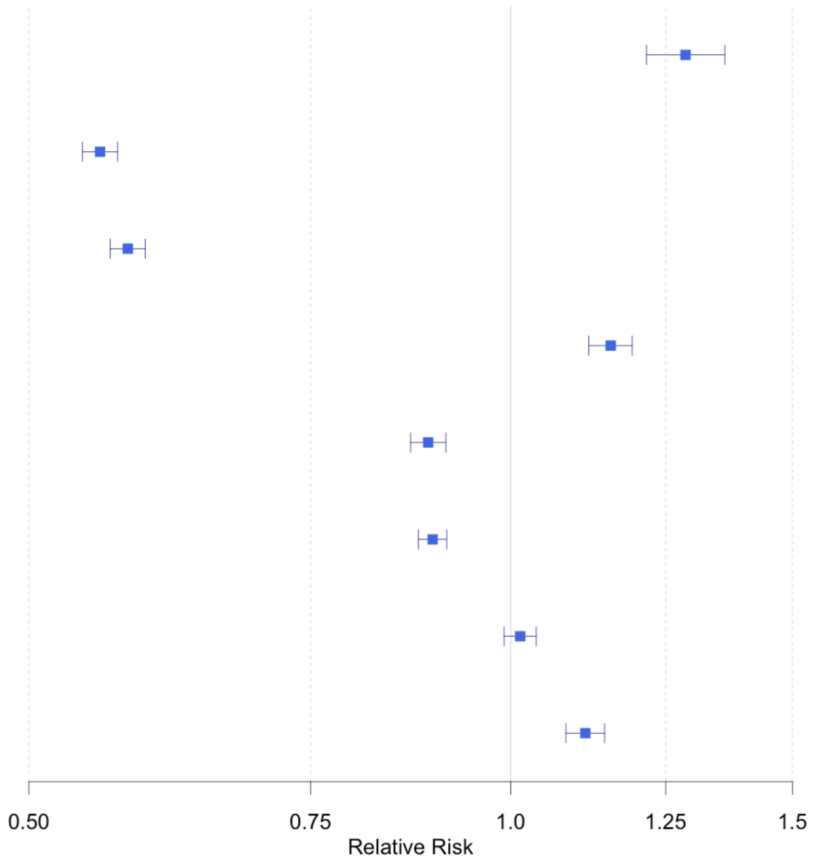

Figures S6-S11. Maps presenting the estimated random effects from the Bayesian spatiotemporal dynamics models used in this analysis. We present the estimated spatiotemporal random effects from the following 6 models: Model 1 “Base Model”, Model 1

“Add SDoH”; Model 2 “Base Model”, Model 2 “Add SDoH”; and Model 3 “Base Model”, Model 3 “Add SDoH”. These models used data from 2005 to 2019, with predictions for 2020 and 2021.

Notice that because we assume that in Model 1 (both the Base and Add SDoH models) the spatial structure is independent of the temporal structure, the estimated spatial random effects are the same for every year.

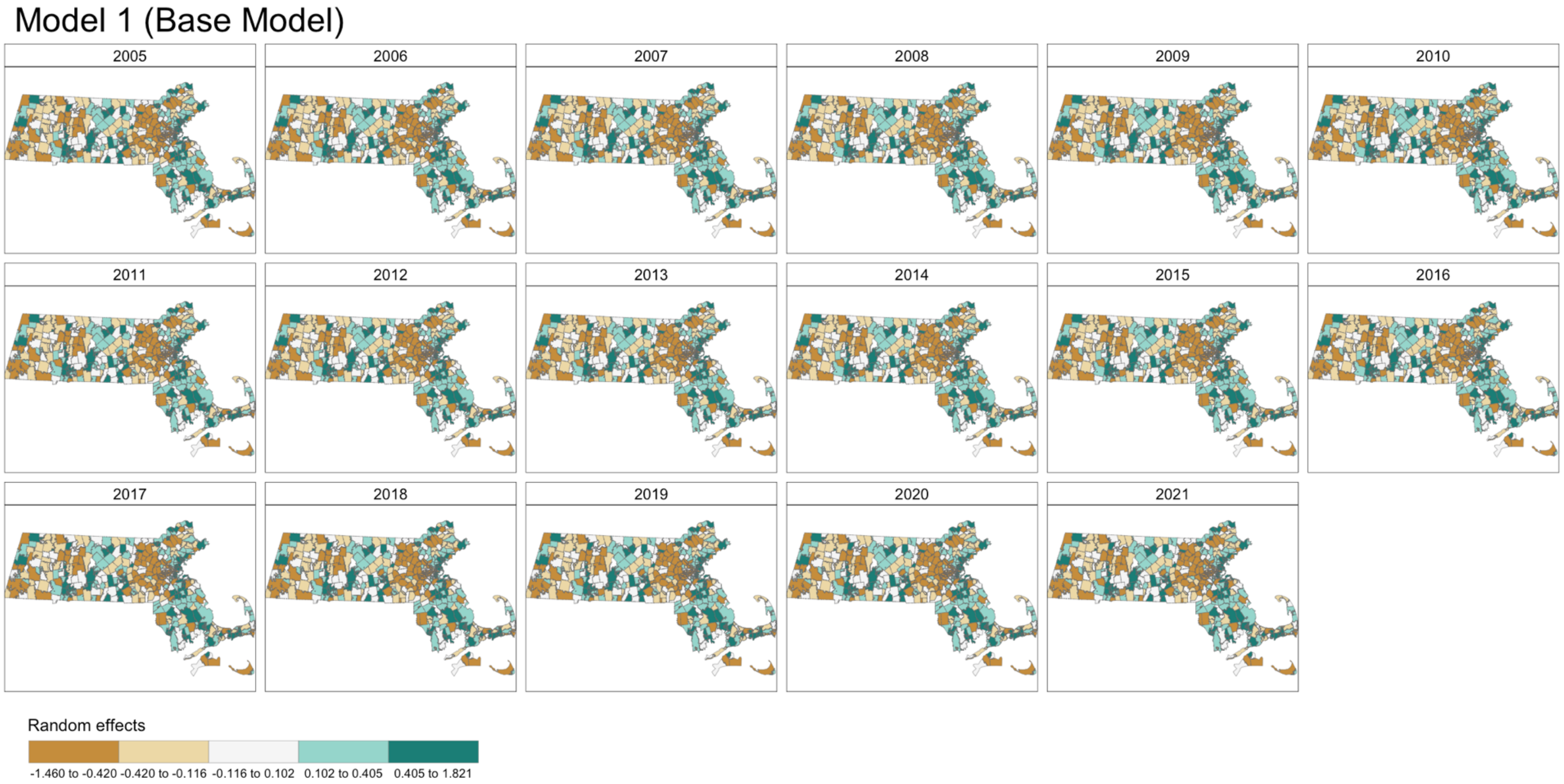

Model 1 (Add SDoH)

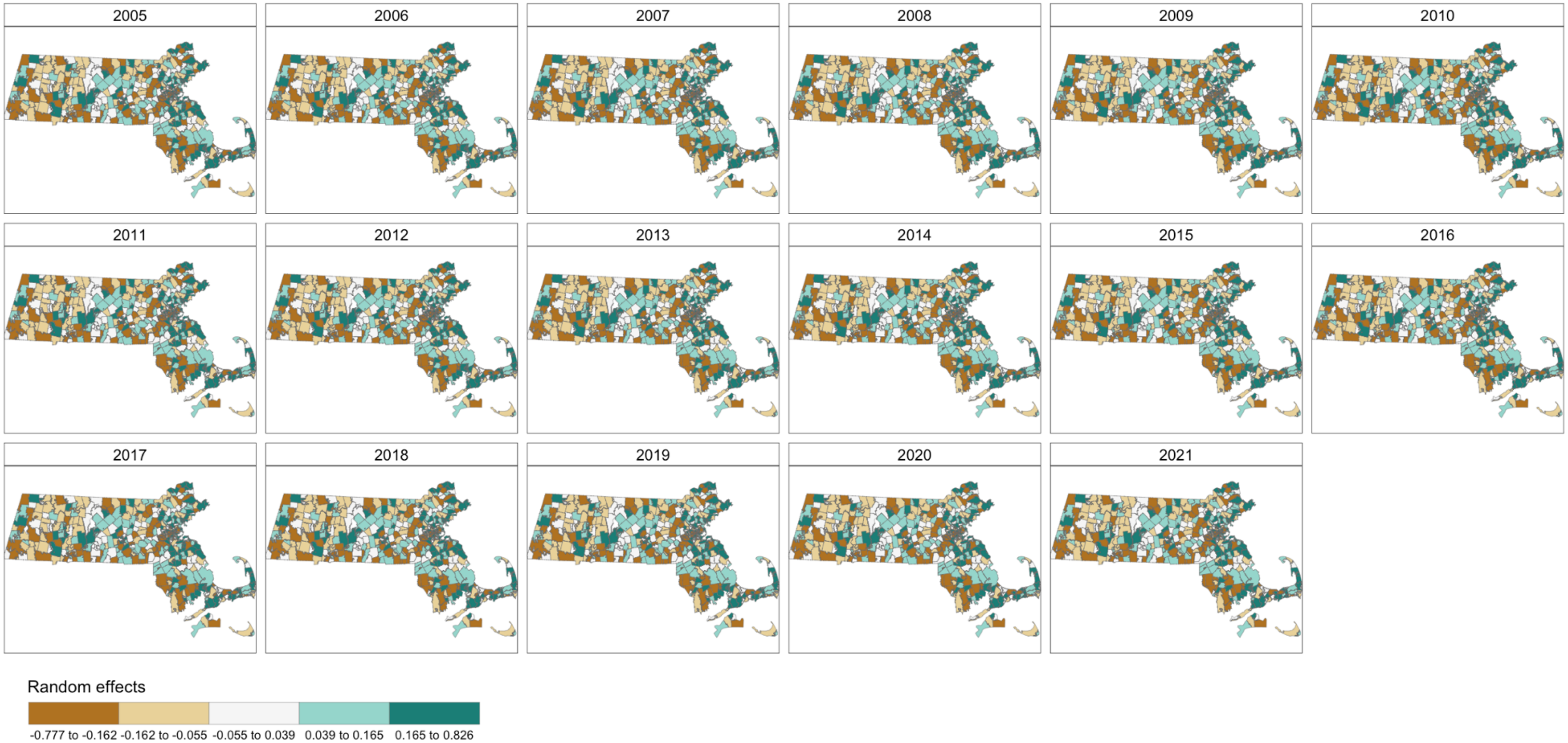

Model 2 (Base Model)

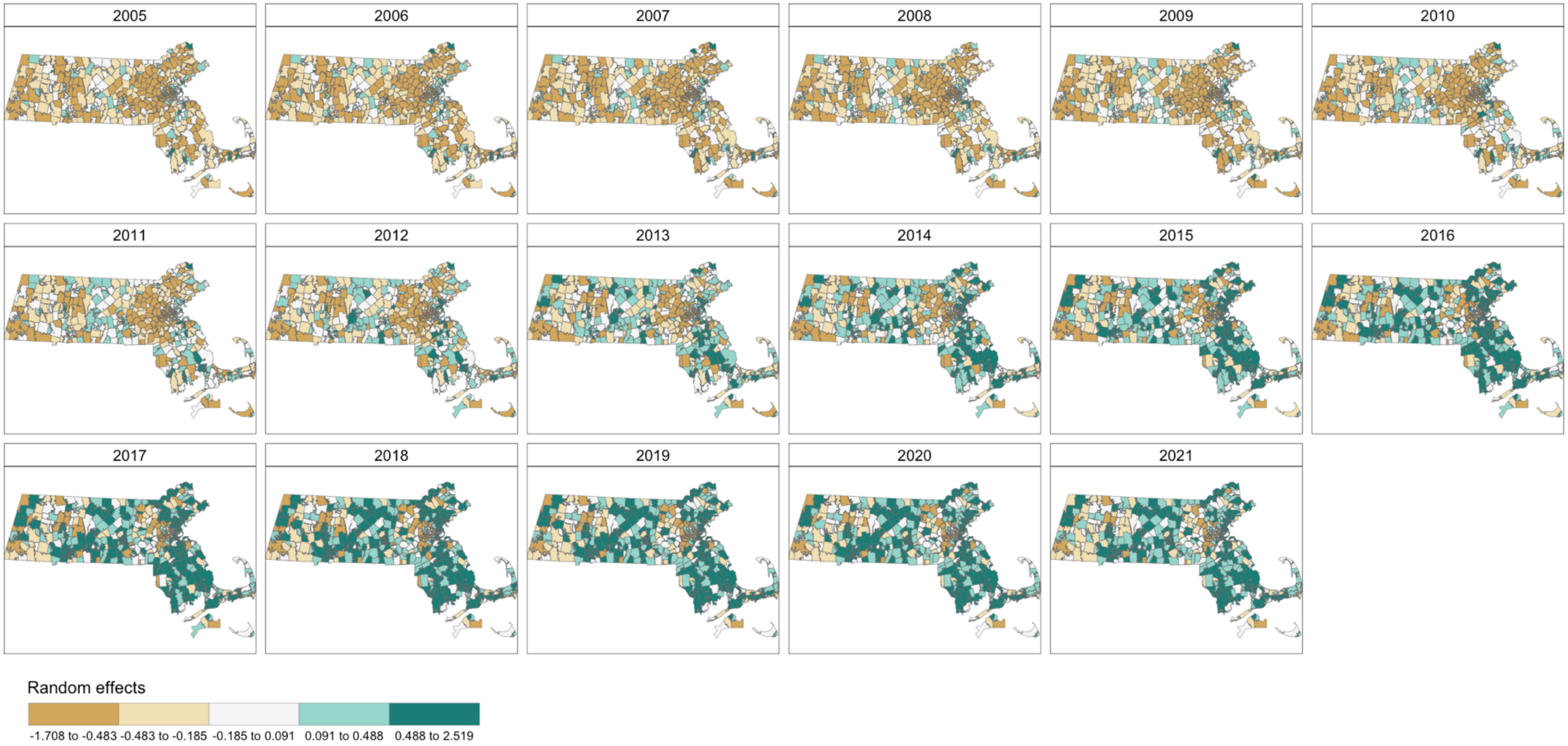

Model 2 (Add SDoH)

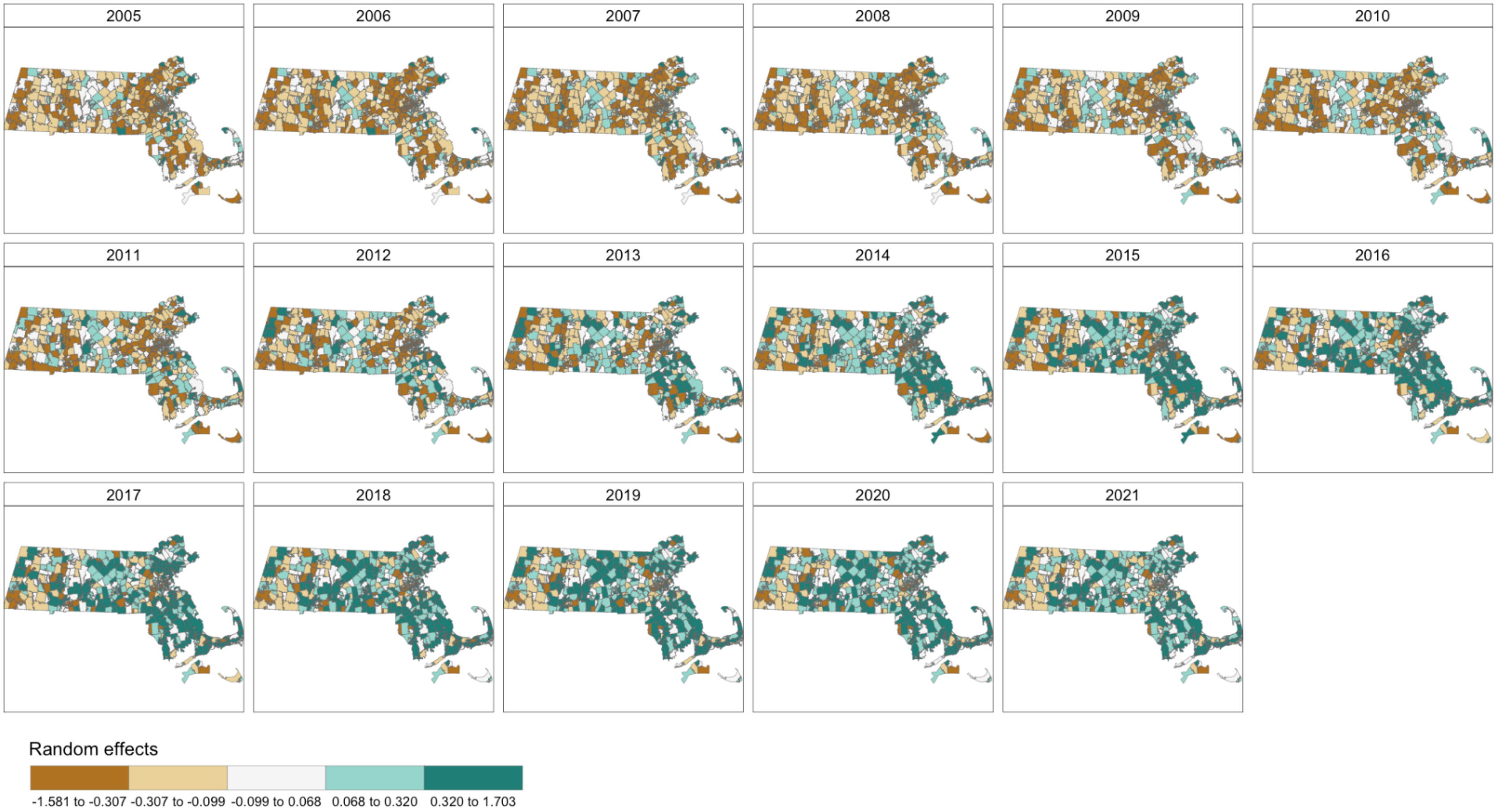

Model 3 (Base Model)

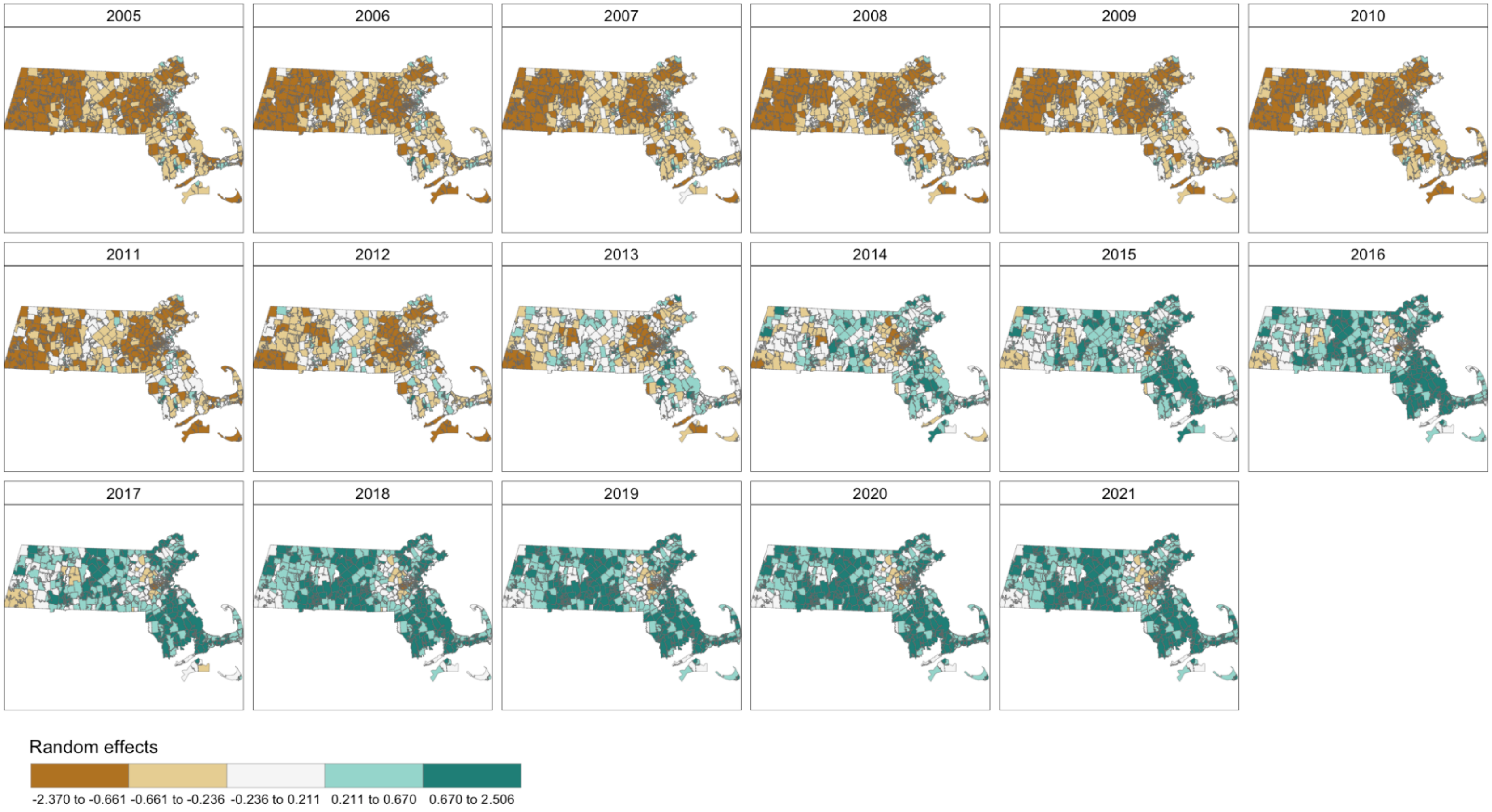

Model 3 (Add SDoH)

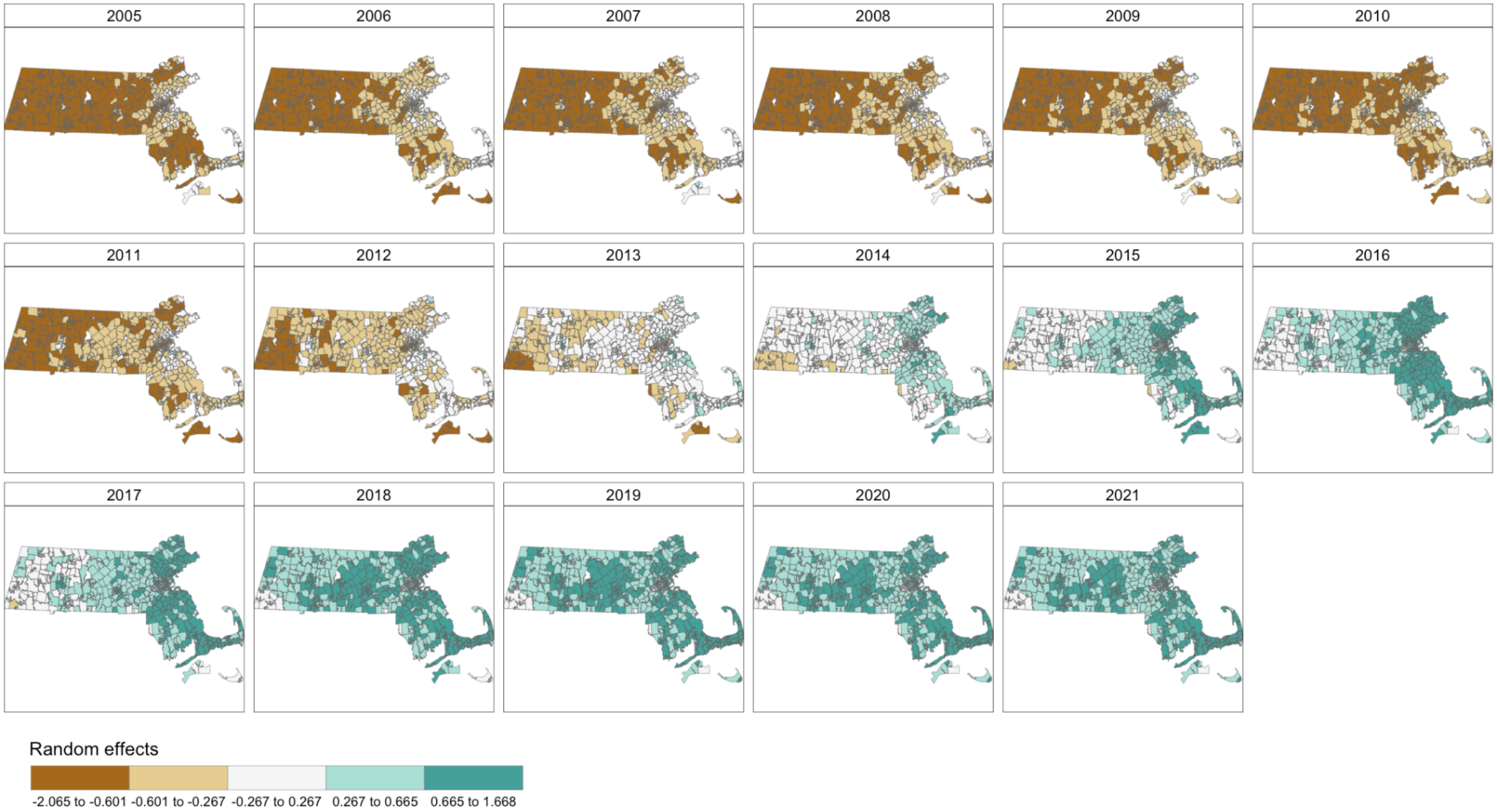

Figure S12. Estimated temporal random effects in selected areas from the Bayesian spatiotemporal dynamics models used in this analysis. We present the estimated spatiotemporal random effects from the following 6 models: Model 1 “Base Model”, Model 1 “Add SDoH”; Model 2 “Base Model”, Model 2 “Add SDoH”; and Model 3 “Base Model”, Model 3 “Add SDoH”. These models used data from 2005 to 2019, with predictions for 2020 and 2021 (with grey shaded background).

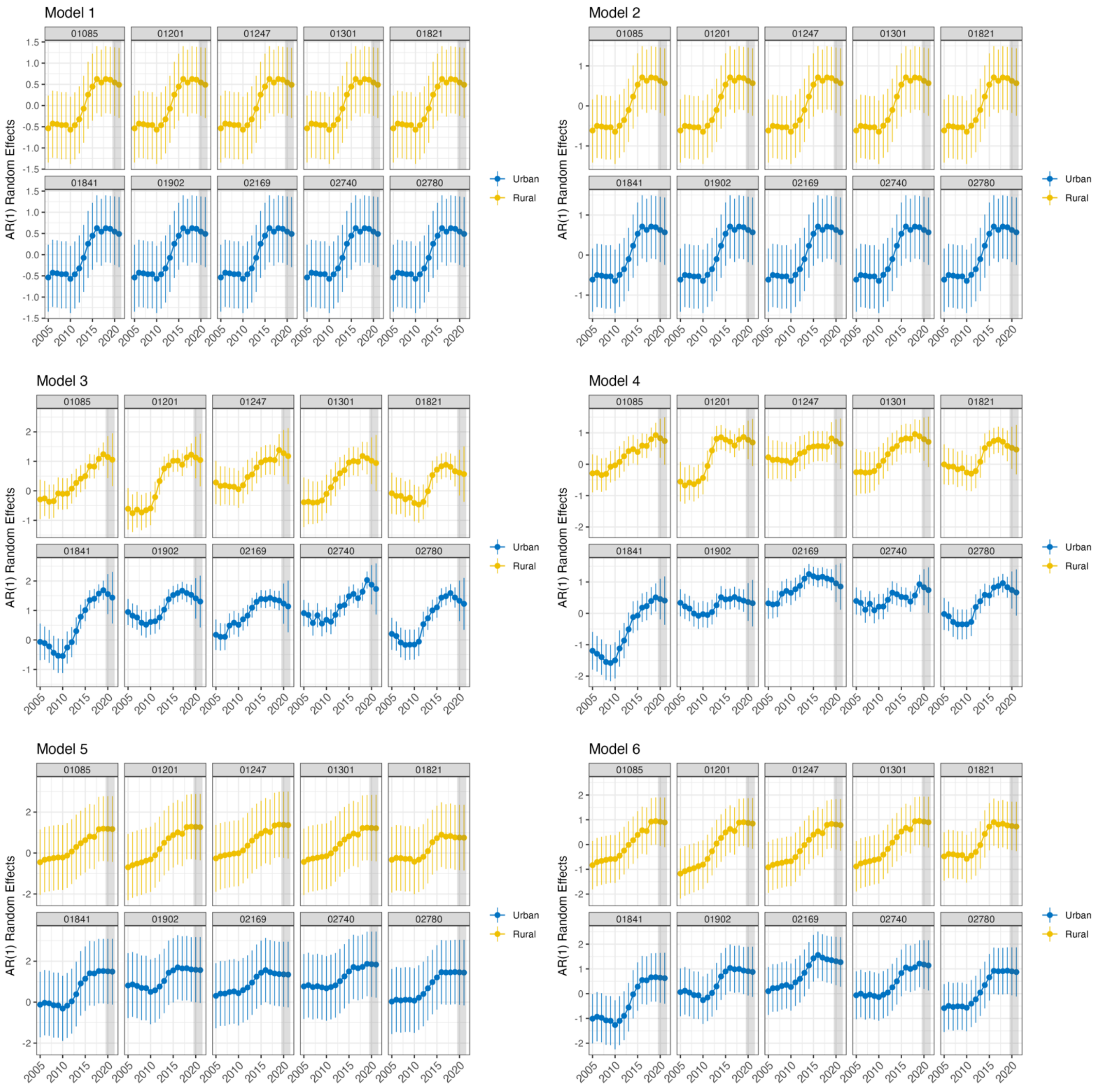

Table S2. Summary of hyperparameters from fitting the proposed Bayesian dynamic spatiotemporal models.

			Posterior Median	0.025quant	0.975quant
Model 1	Base	Precision (Spatial)	2.73	2.32	3.23
		Precision (Temporal)	5.49	1.66	14.13
		Rho	0.93	0.79	0.99
	Add SDOH	Precision (Spatial)	10.91	8.70	13.63
		Precision (Temporal)	4.95	2.61	8.99
		Rho	0.92	0.80	0.97
Model 2	Base	Precision (Spatial)	1.54	1.36	1.74
		Rho (Group)	0.92	0.91	0.93
	Add SDOH	Precision (Spatial)	2.98	2.62	3.40
		Rho (Group)	0.89	0.87	0.91
Model 3	Base	Precision (Spatial)	1.02	0.86	1.21
		Rho (Group)	0.99	0.99	0.99
	Add SDOH	Precision (Spatial)	3.07	2.44	3.87
		Rho (Group)	0.97	0.95	0.98
